# A Study on Fatigue State Evaluation of Rail by the Use of Ultrasonic Nonlinearity

**DOI:** 10.3390/ma12172698

**Published:** 2019-08-23

**Authors:** Bo Zhu, Jaesun Lee

**Affiliations:** 1 Department of Quality and Technology, Shandong Special Equipment Inspection Institute Co., Ltd., Shandong 250101, China; 2 School of Mechanical Engineering, Changwon National University, Changwon 51140, Korea

**Keywords:** railway, stress fatigue, ultrasonic nonlinear wave, wave mixing

## Abstract

Nonlinear ultrasonic testing has been accepted as a promising manner for evaluating material integrity in an early stage. Stress fatigue is the main threats to train safety, railways examinations for stress fatigue are more significant and necessary. A series of ultrasonic nonlinear wave experiments are conducted for rail specimens extracted from railhead with different degree of fatigue produced by three-point bent loading condition. The nonlinear parameter is the indicator of nonlinear waves for expressing the degree the fatigue. The experimental results show that the sensitivity of a third harmonic longitudinal wave is higher than second harmonic longitudinal wave testing. As the same time, collinear wave mixing shows strong relative with fatigue damages than a second longitudinal wave nondestructive testing (NDT) method and provides more reliable results than third harmonic longitudinal waves nonlinear testing method.

## 1. Introduction

Rails are an important element in railway systems. Maintenance of railway systems today is more important and necessary as traffic volume increases. Various types of defects occur in rails, of which manufacturing defects, improper operation, and rolling contact fatigue are key components of the rail defect development process [1]. Railways are mass transit systems that will cause many casualties and massive economic losses. These accidents can arise from various deficiencies in the railway system, and in particular rail defects are a significant threat to the safe operation of rail transport. Most of the rail condition evaluation uses a method of measuring the position change such as external shape measurement or distortion using a laser. However, this method can measure only the surface shape and can’t evaluate the internal defect and the state. Ultrasonic wave velocity and wave attenuation are the most basic material state evaluation methods. However, such a linear ultrasound parameter is insufficient to evaluate microscopic damage. Nonlinear techniques are useful for diagnosing defects at the initial stage and for micro-defects based on signal changes in the frequency domain [2]. Nonlinear ultrasonic test methods mainly use harmonic or quasi-harmonic components [3]. There is an ultrasonic mixing technique in which ultrasonic waves having different center frequencies are mixed to generate a new center frequency ultrasonic wave. Ultrasonic mixing techniques include bulk mixing [2,3,4,5,6,7,8,9] and guided wave mixing [10,11,12,13,14]. Ultrasonic nonlinearities measured through frequency spectrum analysis of received signals include system and material nonlinearities. Experimental systems such as wave generators, amplifiers, transducers and couplants in the experiments will result in strong system nonlinearities [2]. Therefore, reducing system non-linearity is necessary to obtain reliable results. The noncontact ultrasonic method is proposed by several researchers to reduce the uncertainty of the contact condition of a transducer [15,16]. Croxford et al. [9] noted that the wave mixing method is advantageous in reducing the nonlinearity of the system.

An initial theory study to utilize this ultrasonic mixing technique for material condition monitoring is described in Jones et al. [17] and Taylor et al. [18], and numerical simulations have been performed on experimental studies and ultrasonic mixing in various research areas [2,3,4,5,6,7,8,9,10,11,19,20,21]. The main threat of stress fatigue and rail safety is a kind of microstructural defect caused by internal stresses, and the degree of fatigue defects can be represented by ultrasonic nonlinear parameters. The relationship between nonlinearity and fatigue lifetime and fatigue life is expressed in [12,13,22]. There are several studies that have applied various nonlinear techniques to monitor the state of mechanical materials. To compare and analyze the characteristics of each technique, many earlier researchers conduct a nonlinear study using higher harmonic, sub-harmonic ultrasonic nonlinearity and ultrasonic mixing technique. Also, it is necessary to study on early stage damage detection technique to diagnose the fatigue condition of railway rail.

In this study, resonance conditions of high-order harmonic generation and collinear wave mixing are expressed. Experimental studies performed based on a general nonlinear ultrasound method, such as second and third harmonic longitudinal wave tests and collinear wave mixing on the fatigue rail specimen treated with a 3-point cyclic bending load. The main idea of this research paper is comparison between second and third harmonic nonlinear parameter measurement and collinear mixing nonlinear parameter measurement. Even though there are much research was conducted on an ultrasonic nonlinear parameter, it is rarely can find the comparison of each method on the same specimen. Therefore, the experimental comparison results from the fatigued rail specimen are presented in this paper.

## 2. Nonlinear Wave Theory

### 2.1. High-Order Harmonic Nonlinear Waves

**Figure 1:**
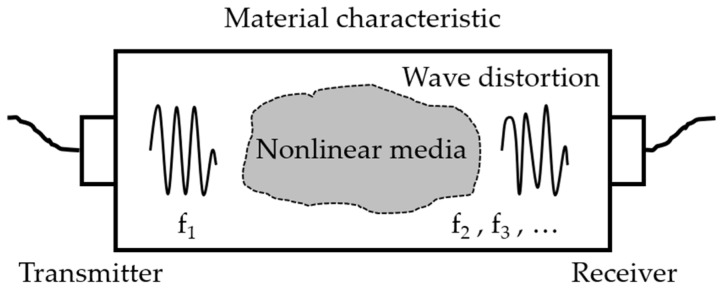
Wave propagation in nonlinear media.

**Figure 2:**
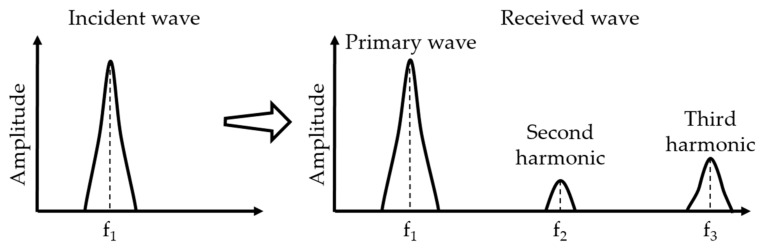
Spectrum changes through nonlinear media.

The physical effects of nonlinear ultrasonic technique are the interaction between the incident wave and material leads to wave distortion (see Figure 1) and generation of the corresponding higher-order harmonic waves (see Figure 2). 

Assume that a one-dimensional model, single frequency longitudinal wave propagates without attenuation, for a small deformation, wave equation can be express by Equation (1) [23]:

(1) $$\frac{\partial \sigma }{\partial x}=\rho \frac{\partial^{2}u}{\partial t^{2}}$$

where ρ is material density, σ is stress term and u is displacement vector along the x direction. The constitutive equation of nonlinear medium can be described with elastic modulus E in Equation (2) [23]:

(2) σ=Ef(ε)

Power series expansion f(ε) is applied to Equation (3):

(3) $$\sigma =Ef\left(\varepsilon \right)=E\left(\varepsilon +\frac{1}{2}\beta_{1}\varepsilon^{2}+\frac{1}{3}\beta_{2}\varepsilon^{3}+\cdots +\frac{1}{n}\beta_{n}\varepsilon^{n}\right)\approx E\left(\varepsilon +\frac{1}{2}\beta_{1}\varepsilon^{2}+\frac{1}{3}\beta_{2}\varepsilon^{3}\right)$$

where $\beta_{n\;}\left(n=1,\;2,\;3,\cdots \right)$ is a factor indicating the order of nonlinear parameter of the material. Subsequently, substituting Equation (3) into Equation (1), one can be regrouped as Equation (4):

(4) $$C^{2}\frac{\partial^{2}u}{\partial t^{2}}=\frac{\partial f\left(\varepsilon \right)}{\partial x}=\frac{\partial \left(\varepsilon +\frac{1}{2}\beta_{1}\varepsilon^{2}+\frac{1}{3}\beta_{2}\varepsilon^{3}\right)}{\partial x}=\frac{\partial^{2}u}{\partial x^{2}}\left[1+\beta_{1}\frac{\partial u}{\partial x}+\beta_{2}{\left(\frac{\partial u}{\partial x}\right)}^{2}\right]$$

In order to solve this problem, a perturbation method is applied. The displacement u is assumed as Equation (5):

(5) $$u\left(x,y\right)=u_{0}\left(x,t\right)+xu_{1}\left(x,t\right)+x^{2}u_{2}\left(x,t\right)+\cdots +x^{n}u_{n}\left(x,t\right)$$

By equating the terms with the same order, the following Equation (6) can be derived:

(6) $$u\left(x,t\right)=A_{1}cos\left(kx-\omega t\right)-\frac{\beta_{1}}{8}k^{2}A_{1}^{2}xcos2\left(kx-\omega t\right)+\frac{\beta_{2}}{24}k^{3}A_{1}^{3}x\left[cos3\left(kx-\omega t\right)+3cos\left(kx-\omega t\right)\right]$$

where ω is angular frequency, and k is wavenumber. So the amplitude of the second harmonic propagation in the material, $A_{2}$, relative with cos2(kx−ωt) the term, is $\beta_{1}k^{2}A_{1}^{2}x/8$. Similarly, third harmonic propagation in the material, $A_{3}$, relative with cos3(kx−ωt) the term, is $\beta_{2}k^{3}A_{1}^{3}x/24$. Regrouping those two relations, nonlinear parameters can be expressed by amplitude ratio directly in Equation (7):

(7) $$\beta_{1}=\frac{8}{k^{2}x}\frac{A_{2}}{A_{1}^{2}},\beta_{2}=\frac{24}{k^{3}x}\frac{A_{3}}{A_{1}^{3}}$$

Obviously, $\beta_{1}$ and $\beta_{2}$ are in proportion to $A_{2}/A_{1}^{2}$ and $A_{3}/A_{1}^{3}$, respectively. 

### 2.2. Collinear Wave Mixing

**Figure 3:**
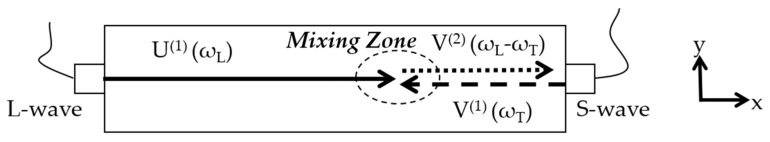
Schematic of collinear wave mixing.

The method is based on the fact that a resonant wave might be generated by two incident waves if resonant conditions are satisfied. The acoustic nonlinear parameter $\beta_{T}$ is the amplitude ratio of primary incident waves and receiving the resonant wave introduced from [5]. The dominant wave mixing technique is that experimental nonlinearity contains less system nonlinearity than conventional higher-harmonic longitudinal ultrasonic testing. Because the received wave obtained from the wave mixing zone of the internal material directly and the subtracting frequency term can eliminate system nonlinearity produced by two incident harmonic waves through measurement system. Hence, the total system nonlinearity is reduced and reliable experimental results are provided [8,9]. The mixed resonant shear wave $V^{\left(2\right)}$ could be generated by a pair of primary longitudinal wave $U^{\left(1\right)}$ and shear wave $V^{\left(1\right)}$ excited in the opposite direction. Here, the resonant shear wave propagation direction is contrary to primary incident shear wave, which called as two-way wave mixing [4,5] see Figure 3.

The two-way wave mixing with subtracting frequency term is conducted in Equation (8):

(8) $$L\left(\omega_{L}\right)+T\left(\omega_{T}\right)\to T\left(\omega_{L}-\omega_{T}\right)$$

And, the primary excited longitudinal and shear waves are defined by Equations (9) and (10):

(9) $$U^{\left(1\right)}=Asin\left(k_{L}x-\omega_{L}t\right)$$

(10) $$V^{\left(1\right)}=Bsin\left(k_{T}x+\omega_{T}t\right)$$

Nonlinear wave equations are used for the description of waves propagating in nonlinear media, Goldberg expressed the nonlinear wave Equations (11)–(13) as below:

(11) $$u_{tt}-C_{L}^{2}u_{xx}=\left(3C_{L}^{2}+C_{111}/\rho \right)u_{x}u_{xx}+\left(C_{L}^{2}+C_{166}/\rho \right)\left(v_{x}v_{xx}+w_{x}w_{xx}\right)$$

(12) $$v_{tt}-C_{T}^{2}v_{xx}=\left(C_{L}^{2}+C_{166}/\rho \right)\left(u_{x}v_{xx}+v_{x}u_{xx}\right)$$

(13) $$w_{tt}-C_{T}^{2}w_{xx}=\left(C_{L}^{2}+C_{166}/\rho \right)\left(u_{x}w_{xx}+w_{x}u_{xx}\right)$$

The nonlinearity parameter is defined as $\beta_{T}=-\;\left(C_{L}^{2}+C_{166}/\rho \right)$. Substituting primary longitudinal and shear waves equations into the governing Equations (11)–(13), one can be derived in Equation (14):

(14) $$\begin{array}{ll} v_{tt}^{\left(2\right)}-C_{T}^{2}v_{aa}^{\left(2\right)}=\frac{1}{2} & \beta_{T}ABk_{L}k_{T}\left(k_{L}-k_{T}\right)\sin\left[\left(k_{L}-k_{T}\right)x-\left(\omega_{L}+\omega_{T}\right)t\right]\\ & +\frac{1}{2}\beta_{T}ABk_{L}k_{T}\left(k_{L}+k_{T}\right)\sin\left[\left(k_{L}+k_{T}\right)x-\left(\omega_{L}-\omega_{T}\right)t\right] \end{array}$$

Assume that the receiving resonant shear wave is Equation (15),

(15) $$V^{\left(2\right)}=Csin\left(\left(k_{L}+k_{T}\right)x-\left(\omega_{L}-\omega_{T}\right)t\right)$$

where C is the amplitude of the mixed wave demonstrated by Equation (16):

(16) $$C=\frac{1}{2}\beta_{T}AB\frac{\omega_{T}\left(C_{T}\omega_{L}+C_{L}\omega_{T}\right)}{\left(C_{L}+C_{T}\right)\left(C_{T}\omega_{L}+2C_{L}\omega_{T}-C_{L}\omega_{L}\right)}$$

Obviously, amplitude C in Equation (16) is in proportion to the primary waves’ frequencies. For the resonant condition, meaning that the denominator equals zero and the mixed wave amplitude approaches to infinity. Therefore, the frequency relation of the excited waves can be described as follows Equation (17):

(17) $$\frac{\omega_{T}}{\omega_{L}}=\frac{C_{L}-C_{T}}{2C_{L}}$$

## 3. Experimental Setup

**Figure 4:**
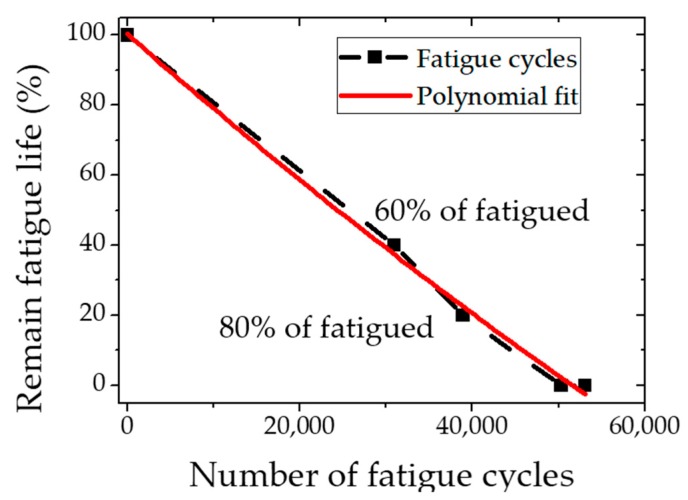
Fatigue life of KR 60 rail specimens.

KR 60 rails are widely used in Korean transport systems. The material properties are listed in Table 1 [24]. The specimen used in this study is a rectangular bar of 350 × 30 × 60 mm^3^ in rail head. Three-point bending fatigue tests were performed on the specimens. The load was 12 ton and the fatigue cycle was 2.5 Hz. In order to measure the fatigue life of the specimen, the fatigue cycle was measured by using two control specimens up to failure was about 51,700 cycles on average. Based on the fatigue life curve as depicted in Figure 4, fatigue test specimens of 60% and 80% were prepared based on the life span of undamaged test specimens and fatigued specimen.

**Figure 5:**
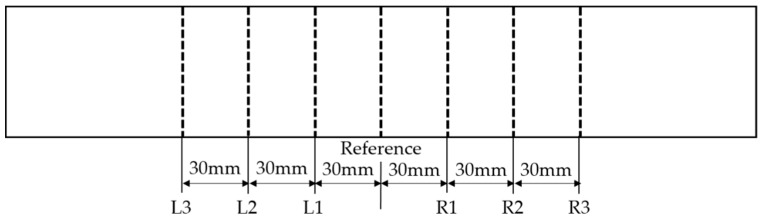
Measurement points of the specimen.

High-order harmonic longitudinal wave and wave mixing tests are applied to seven detection points defined at the same distance in the central region (see Figure 5). Use the high-power tone burst system to generate narrowband signals at seven detection points. The transducer is mounted on the specimen with couplants and consistent pressure to ensure constant contact conditions. Signal processing uses the Fast Fourier Transform (FFT) to obtain the spectrogram.

### 3.1. High-Order Harmonic Longitudinal Wave Nonlinear Testing

**Figure 6:**
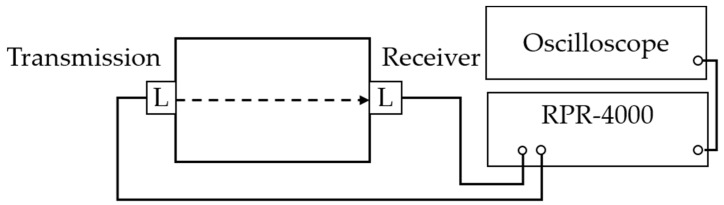
Schematic of higher harmonic longitudinal wave nonlinear testing.

The experimental setup for measuring higher order harmonic nonlinearities is shown in Figure 6. Two longitudinal wave transducers are located in a face to face each other throughout the specimen. One transducer works as transmission and the other is working as a receiver. The letter L denote longitudinal wave transducer in the Figure 6. High-voltage tone burst system calls such as the RPR-4000 (Ritec Inc., Warwick, RI, USA) have been working to generate 20 cycles with the PZT sensor (Olympus NDT NE Inc., Quebec City, QC, Canada) signal at 5 MHz. The frequency bandwidth can be easily limited by the window tone burst signal generated by the measurement system.

To detect the corresponding higher-order harmonic frequency components primarily, the bandwidth of the receiving transducer should cover the frequency range. In the spectrum of the signal, it can be found that second harmonic wave at the quadratic frequency at 10 MHz and third harmonic wave at the triple frequency at 15 MHz. The sensor conditions are listed in Table 2. 

### 3.2. Wave Mixing Nonlinear Testing

Once satisfying the resonant condition, the excited frequency relation can be derived by substituting wave speeds terms into Equation (17). Where, theoretical longitudinal and transverse waves’ speeds are $C_{L}=5856.4\;m/s$ and $C_{T}=3130.4\;m/s$, respectively, calculated from [25,26]. One can be expressed by Equation (18):

(18) $$\frac{\omega_{T}}{\omega_{L}}=\frac{C_{L}-C_{T}}{2C_{L}}=\frac{5856.4-3130.4}{2\times 5856.4}\approx 0.233$$

One can be simplified as Equation (19),

(19) $$U^{\left(1\right)}\left(\omega_{L}\right)-V^{\left(1\right)}\left(0.233\omega_{T}\right)=V^{\left(1\right)}\left(0.767\omega_{L}\right)$$

**Figure 7:**
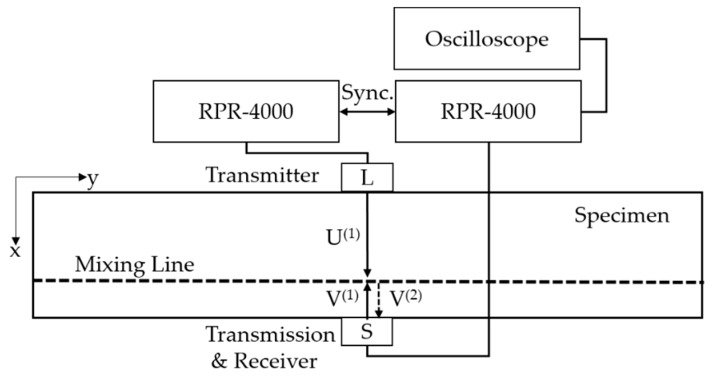
Schematic of wave mixing experimental setup.

The schematic of wave mixing experimental setup is shown in Figure 7. Two transducer are located in a face to face each other throughout the specimen. Longitudinal wave transducer works as transmitter and shear wave transducer is working as a transmitter and receiver. The letter *L* and *S* denote longitudinal and shear wave transducer in Figure 7, respectively. Two tone burst devices RPR-4000 are connected to each other and synchronized for transmitting and receiving waves. The oscilloscope is used for showing and validating the received signals. In order to fully collect data, a sampling rate of 125 MHz was chosen.

A 2.5 MHz excited transverse wave and a 10 MHz excited longitudinal wave were meet in the internal specimen to produce a mixed ultrasonic wave at 7.5 MHz. Sensors information are listed in Table 3.

It should be noticed that due to the difference in wave speed, the mixing line is close to the bottom shear transducer. The mixing time *T* is 6.676 μs, which can be calculated directly by the equation $\left(C_{T}+C_{L}\right)T=L$, where, L is the height of the specimen equal to 60 mm. Obviously, the distance between mixing lines and the shear transducer is about 20 mm as depicted in in Figure 7. Certainly, it is feasible for defining a mixing line in the random position with time delay.

## 4. Experimental Results

### 4.1. High-Order Harmonic Longitudinal Wave Nonlinear Testing

**Figure 8:**
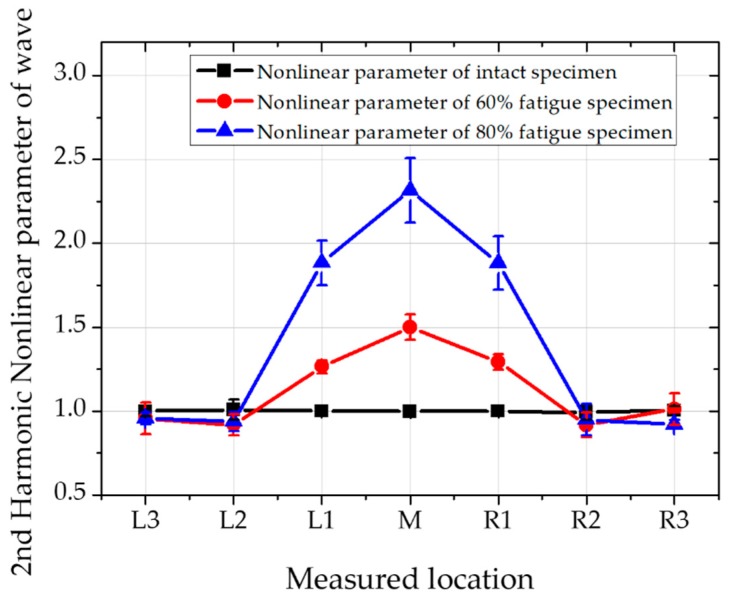
Second harmonic wave for measuring nonlinear parameter at various positions.

**Figure 9:**
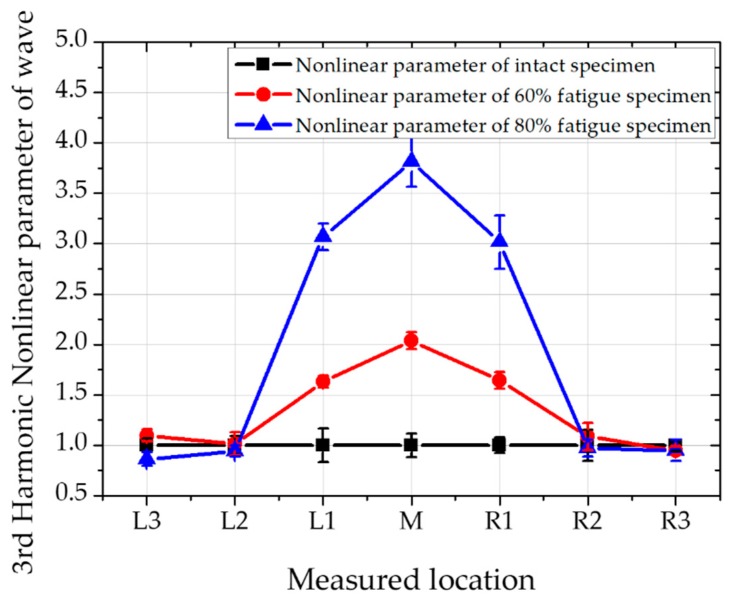
Third harmonic wave for measuring nonlinear parameter at various positions.

The signal of higher-order harmonic longitudinal waves can be received by receiving transducers and stored by RPR-4000 equipment. After signal processing, the corresponding spectrum can be easy obtained. The second and third nonlinear value can be described by amplitude ratio of second harmonic and third harmonic wave amplitude to primary wave amplitude, respectively. Relative second and third harmonic nonlinear parameter for application is defined by $\beta_{1}\propto A_{2}/A_{1}^{2}$ and $\beta_{2}\propto A_{3}/A_{1}^{3}$, respectively. The normalized nonlinear parameter value at each detecting point is shown in Figure 8 and Figure 9 for second and third order harmonic longitudinal wave, respectively.

The higher harmonic nonlinear parameters are compared as seen in Table 4. The ratio of nonlinear parameters is listed at each of the measured points. At the fatigue status, the nonlinear parameters at third harmonics are generally bigger than second harmonics. Interestingly, at the measured point of L1 and R1, the ratio of second and third harmonic nonlinear parameters become greater at 80% of fatigue status than 60%. Therefore, it can be assumed that as fatigue cycles are increased, third harmonic nonlinearity becomes sensitive than second harmonics

### 4.2. Collinear Wave Mixing Nonlinear Testing

**Figure 10:**
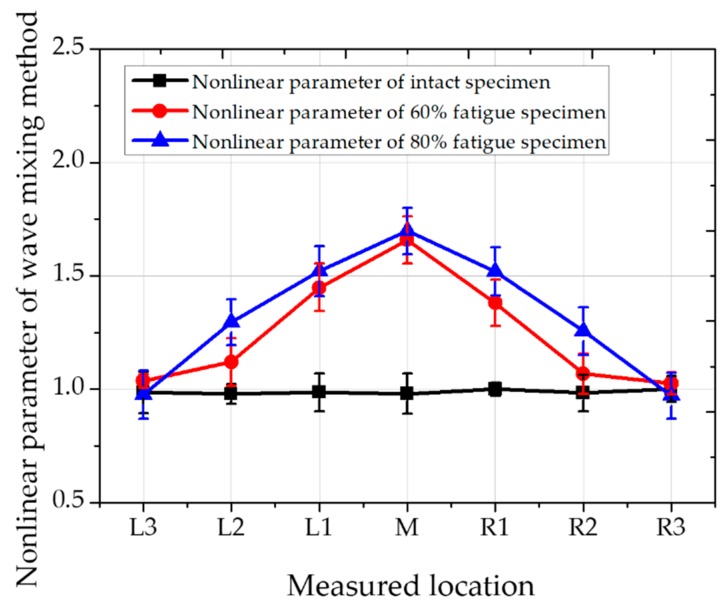
Wave mixing for measuring nonlinear parameter at various positions.

Butterworth filter is applied for signal processing. Finally, the spectrum of collinear wave mixing can be obtained. In order to measure the material nonlinearity of the rail specimens, the amplitude of the new generated shear wave $V^{\left(2\right)}$ was normalized to the product of the two incident amplitudes $U^{\left(1\right)}$ and $V^{\left(1\right)}$ measured in volts [9]. Figure 10 shows the normalized nonlinear parameter values of the detecting points [27].

## 5. Conclusions

A feasibility study is carried out for higher harmonic longitudinal tests and collinear mixed wave nonlinear tests to demonstrate fatigue rail specimens. Three different stress fatigue test specimens are extracted from the rail head and subjected to three point cyclic bending loading. The theoretical analysis focuses on the phenomenon of wave distortion and the generation of new harmonics when waves propagate in a nonlinear medium. Experiments are performed on fatigue test specimens based on high order longitudinal test and wave mixing test methods. The value of the nonlinear parameter at the detection point is used to indicate the status of fatigue. Nonlinear trends are clear by normalizing the value of a nonlinear parameter normalized to an undamaged specimen. In general, the nonlinearity is proportional to stress fatigue.

The nonlinear parameter values of the third harmonic and second harmonic in the central region are about 3 ~ 4 times and 1.5 ~ 2.5 times higher than the undamaged sample, respectively. In other words, the third harmonic longitudinal nonlinear test is more sensitive than the second harmonic longitudinal nonlinear test. It should be noted that higher order harmonics represent low energy transfer reflected by the experimental operation and the value of the wave amplitude which is heavily influenced by the experimental system. Therefore, the accuracy of the third harmonic nonlinearity can’t be guaranteed. Although second harmonic nonlinear parameter measurement is popular and widely used for micro defect inspection, third harmonic nonlinear parameter measurement provide an alternative wave for stress fatigue measurement. In this research we conduct a feasibility study on 3rd harmonic nonlinear parameter measurement for fatigue on a rails.

The nonlinearity of the wave mixing method for these fatigue test specimens is much clearer and more remarkable than the second harmonic longitudinal test method, and the value of the nonlinear parameter at the center portion is lower than that described later. The advantage of third harmonic nonlinear parameter measurement and wave mixing nonlinear method is clearly can see in the results by comparing L2 and R2 values in Figure 8, Figure 9 and Figure 10. It is obvious that the maximum nonlinear value appears in the maximum deformed location, central location of the specimen. However, the measured location is getting further from the central location, only wave mixing technique can clearly differentiate the status of fatigue through the specimen. Obviously, wave mixing is more accurate by eliminating nonlinearities in the system.

This study validates the probabilities of nonlinear techniques for fatigue failures and provides quantitative comparisons between three common nonlinear techniques. High-order harmonics and wave mixing wave is sensitive to microstructural defects, so you can draw conclusions, but the results of wave mixing are more reliable.

## Figures and Tables

**Table 1:** Chemical composition and material properties of KR 60.

Chemical Composition (%)					Mechanical Properties		
Carbon	Silicon	Manganese	Phosphorus	Sulfur	Tensile strength (MPa)	Elongation (%)	Hardness (HBW)
0.63 ~ 0.75	0.15 ~ 0.3	0.7 ~ 1.10	<0.03	<0.025	>880	>10	260 ~ 300

**Table 2:** Experimental conditions for higher harmonic measurement technique.

Transducer	Sensor Type	Wave Type	Frequency
Transmitter	Longitudinal wave	Primary wave	5 MHz
Receiver	Longitudinal wave	Second harmonic wave	10 MHz
	Longitudinal wave	Third harmonic wave	15 MHz

**Table 3:** Experimental conditions for wave mixing technique.

Transducer	Sensor Type	Wave Type	Frequency
Transmitter	Longitudinal wave	Primary wave	10 MHz
	Shear wave	Primary wave	2.5 MHz
Receiver	Shear wave	Mixed wave	7.5 MHz

**Table 4:** Comparison on second and third harmonic nonlinear parameter.

	Measured Point	L3	L2	L1	M	R1	R2	R3
Test Specimen								
Fatigue Status	Ultrasonic Nonlinear Parameter							
60%	Second harmonic parameter	0.95628	0.910762	1.26546	1.504283	1.29456	0.922689	1.009529
	third harmonic parameter	1.09837	1.01397	1.63064	2.0374	1.64136	1.08693	0.94794
	Ratio (3rd/2nd)	1.148586	1.11332	1.288575	1.354399	1.26789	1.178003	0.938992
80%	Second harmonic parameter	0.95707	0.929836	1.88444	2.31871	1.8838	0.955423	0.918632
	third harmonic parameter	0.86211	0.94393	3.06951	3.81586	3.01615	0.9726	0.95062
	Ratio (3rd/2nd)	0.900781	1.015158	1.628871	1.645682	1.601099	1.017978	1.034821
